# Systems biology can provide guidance to synthetic biology in the pursuit of new drug targets

**DOI:** 10.3389/fphar.2026.1770107

**Published:** 2026-03-26

**Authors:** Eberhard O. Voit

**Affiliations:** Department of Biological Sciences, The University of Texas at Dallas, Richardson, TX, United States

**Keywords:** computational model, drug targeting, dynamics, metabolic pathway, systems analysis, β-lapachone

## Abstract

Many diseases are caused by an elevated or decreased level of some metabolite but it is not always *a priory* clear which process or processes associated with the metabolite could or should be altered pharmaceutically to effect a return to normalcy. With a case study, this perspective article demonstrates the power of systems biological analysis for guiding drug targeting. The case study analyzes a seemingly simple linear metabolic pathway whose end product triggers the activation of a transcription factor that controls the gene coding for an enzyme catalyzing an upstream step within the pathway. This physiological feedback mechanism might seem artificial but constitutes a type of circuit observed in physiology and pathology. For example, the illustration pathway is a simplified version of a cancer-related redox circuit involving the enzyme NQO1 (NAD(P)H:quinone oxidoreductase 1), which is protective against oxidative stress. The circuit can be pharmaceutically manipulated with the anti-cancer agent β-lapachone, which induces apoptosis through the generation of reactive oxygen species and thereby inhibits tumor growth. The catalysis of β-lapachone by NQO1 results in the generation of hydrogen peroxide, which activates a transcription factor controlling the synthesis of NQO1, thereby closing a positive feedback loop. The analysis of a simplified version of this scenario demonstrates how a physiological circuit can lead our intuition astray. Dynamic modeling easily overcomes this challenge and thereby offers a powerful exploratory tool for the targeted design of pharmaceutical interventions. It permits reliable explanations and prescriptions for feasible solutions that might be implemented with methods of synthetic biology.

## Introduction

The fusion of pharmacology and synthetic biology has enormous potential for the next phase of drug development. It will synergistically combine the efforts of the new subfield of Quantitative Systems Pharmacology (QSP) (Androulakis, 2016) with the goals of synthetic biology to create novel, biologically inspired therapeutics and redesigned biological circuits in a precisely targeted manner.

Pharmacology has an esteemed history that can be traced back to antiquity. Having largely used methods of chemistry in the past, it is now increasingly making use of computational approaches that shed light on pertinent topics, from quantitative structure-activity relationships (Li et al., 2025) to bioinformatics (Zhang et al., 2025) and molecular modeling with methods of structural biology (Congreve et al., 2005) to predictive dynamic modeling with differential or difference equations (Androulakis, 2016; Davis et al., 2019; Voit, 2002; Aradi and Erdi, 2006; Voit and Kemp, 2025).

It is not easy to identify the roots of synthetic biology. The field clearly draws from genetic and metabolic engineering but has also been spearheading other targeted molecular manipulations, especially, to create artificial molecules that resemble actual biological molecules but have defined, novel roles. Prominently, over 500 therapeutic monoclonal antibodies have been under investigation or approved by regulatory agencies around the world, e.g., for inflammatory and autoimmune diseases (Adalimumab; Humira) (Ellis, 2025) and cancer (Pertuzumab; Perjeta) (Lyu et al., 2022). Also of pharmaceutical interest are aptamers, which are synthetically generated, single-stranded DNA or RNA molecules that mimic antibodies by folding into complex 3D shapes and bind with high specificity and affinity to target proteins or other small molecules (Dunn et al., 2017). Two examples are drugs for age-related neovascular macular degeneration (AMD): Pegaptanib (Macugen), the first FDA-approved aptamer for AMD (Vinores, 2006), which however is no longer available, and the newer drug Izervay (Avacincaptad Pegol) (Shakeel et al., 2024).

Beyond molecules, synthetic biology has made strong progress in creating altered metabolic pathways and physiological circuits. Bacteria were reprogrammed to produce various organic compounds that were foreign to them (Gudmundsson and Nogales, 2021) and even let them see light (Levskaya et al., 2005). New pathways were created in bacteria displaying predictable oscillations (Atkinson et al., 2003; Elowitz and Leibler, 2000). Sophisticated pathway rerouting in yeast led to the production of the malaria drug Artemisinin (Paddon and Keasling, 2014). As Christopher Voigt predicted: “Synthetic biology will transform how we grow food, what we eat, and where we source materials and medicines” (Voigt, 2020).

The challenge for an enhanced role of synthetic biology in pharmacology and drug development is now the *in vivo* implementation of circuits and altered pathways in humans. This transition is all but trivial and will require a transition from the typical stitching together of stretches of *de novo* DNA or DNA from other organisms to altering pathological pathways through targeted genome editing approaches with tools like CRISPR (He et al., 2025; Voit et al., 2023) or with aptamers (Dunn et al., 2017), thereby returning them to quasi-normalcy or at least acceptable functionality.

While it is relatively straightforward to assess the success of an artificially created molecule, the effective, targeted modification of a pathway can be difficult to evaluate, because alterations of regulated pathways can result in unexpected outcomes. The case study in this article illustrates this challenge with a simple-looking pathway that responds to interventions in surprising ways.

**FIGURE 1:**
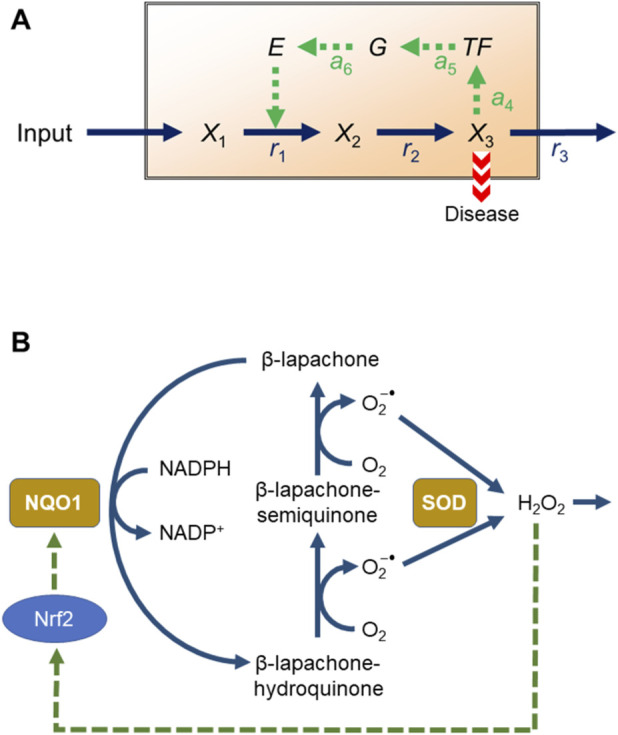
**(A)** Linear metabolic pathway with physiological feedback. *X*_3_ is a risk factor of disease. **(B)** Simplified reaction scheme of β-lapachone catalysis by NQO1, spontaneous production of O_2_^−•^ and H_2_O_2_, and activation of the transcription factor Nrf2, which controls the expression of the gene coding for NQO1. The involvement of NADPH in the generation of O_2_^−•^ is not explicitly shown.

The pathway consists of a linear chain of metabolic reactions, where the end product triggers the activation of a transcription factor responsible for the expression of a gene that codes for an enzyme upstream in the pathway (Figure 1A). This set-up may seem artificial, but its structure is encountered in physiology and pathology. For instance, it directly resembles a pathway involving the cancer drug β-lapachone (Figure 1B) in a simplified manner. We begin with a description of the β-lapachone pathway.

## Motivating example

Cancer cells are often metabolically very active, which creates an environment of elevated levels of reactive oxygen species (ROS), such as superoxide (O_2_^−•^) and hydrogen peroxide (H_2_O_2_) (Aykin-Burns et al., 2009). The cancer cells respond to the resulting oxidative stress by increasing the activity of metabolic pathways that generate reducing power, for instance, through NADPH, and by upregulating antioxidant systems.

One of these antioxidant systems involves the enzyme NQO1 (NAD(P)H:quinone oxidoreductase 1), which plays diverse physiological roles (Ross et al., 2017; Ross and Siegel, 2021). For instance, it is a component of the redox system in the plasma membrane, where it generates antioxidant forms of ubiquinone and vitamin E. It also serves as an mRNA-binding protein that regulates translation and binds to microtubules, for instance in the mitotic spindles. NQO1 is furthermore an efficient intracellular generator of NAD^+^ and protects numerous proteins from proteasomal degradation. Importantly here, NQO1 converts harmful quinones to less harmful hydroquinones in an iterative manner. This process of *redox cycling* generates ROS from quinones, as quinones are repeatedly reduced and then re-oxidized. NQO1 is highly expressed in many solid tumors, which has made it a promising cancer-specific target.

The anti-cancer agent β-lapachone is a quinone that exploits NQO1’s activity in cancer cells through redox cycling (Figure 1B): NQO1 reduces β-lapachone through the oxidation of NADPH into β-lapachone-hydroquinone; the latter spontaneously reacts with oxygen to form the unstable β-lapachone-semiquinone, which again spontaneously reacts with oxygen to return β-lapachone. In the process, both reactions generate the free radical O_2_^−•^ (Ross and Siegel, 2021; Li et al., 2019; Silvers et al., 2017; Raddatz et al., 2023; Lewis et al., 2018). Because β-lapachone is repeatedly reduced and then re-oxidized and thereby returned to its original form, the cycle repeats, continually producing O_2_^−•^. The free O_2_^−•^ radicals cause cell damage by initiating chain reactions that produce other harmful reactive oxygen species. In the cytosol, superoxide dismutase converts some of the O_2_^−•^ into H_2_O_2_, which is also damaging, but less so (Winterbourn, 2013). H_2_O_2_ is subsequently converted to water and oxygen by the peroxiredoxin, glutathione peroxidase, and protein thiol oxidation systems. Intriguingly, H_2_O_2_ activates the transcription factor Nrf2 (Nuclear factor-erythroid 2-related factor 2), which controls the synthesis of NQO1, thereby creating a positive regulatory feedback loop (Ross and Siegel, 2021; Nioi et al., 2003; Covas et al., 2013; Panieri and Saso, 2021; Li et al., 2014) (Figure 1B). Detailed systems analysis has shown that the NQO1 mechanism can display substantial quantitative variations, which may cause strong interpersonal differences in treatment efficiency (Raddatz et al., 2023).

The following illustration of the power of systems biology uses a generic pathway that is reminiscent of the NQO1 redox cycling system, but stripped of much detail to allow a simplified—and therefore clearer—analysis (Figure 1A). Its starting point is a generic metabolite *X*_1_ (reminiscent of β-lapachone), which through an intermediate, *X*_2_ (reminiscent of O_2_^−•^), triggers the production of an end product, *X*_3_ (reminiscent of H_2_O_2_). *X*_3_ activates a transcription factor *TF* (Nrf2), which controls the gene *G* coding for the enzyme *E* (NQO1), which catalyzes a step within the pathway (reduction of β-lapachone).

Deviating from the motivating example, we assume that the level of the generic end product, *X*_3_, is too high and should be lowered through drug intervention.

## Initial model set-up

Before we investigate the model in Figure 1A, it is useful to simplify the pathway even further and study a corresponding linear pathway without the transcription factor *TF* and the gene *G* (Supplementary Section S1). In this simplified scenario, we suppose that a patient has an elevated level of metabolite *X*_3_ that is to be lowered to restore healthy physiology. Considering a generic *Input*, precursors *X*_1_ and *X*_2_, and an enzyme *E* catalyzing the conversion of *X*_1_ into *X*_2_, it is straightforward to formulate a model of mass-action type (Voit et al., 2015), which contains the variables of interest and three rate constants, *r*_1_ – *r*_3_:

$$\frac{dX_{1}}{dt}=Input-r_{1}{\;E\;X}_{1}$$

$$\frac{dX_{2}}{dt}=r_{1}{\;E\;X}_{1}-r_{2}{\;X}_{2}$$ (1)

$$\frac{dX_{3}}{dt}=r_{2}{\;X}_{2}-r_{3}{\;X}_{3}$$

Details are presented in Supplementary Section S1. To permit numerical explorations, we define, more or less arbitrarily, *Input* = 1, *E* = 1, *r*_1_ = 0.8, *r*_2_ = 1.6, *r*_3_ = 0.4. With these rate constants, the system has a steady state of (*X*_1ss_, *X*_2ss_, *X*_3ss_) = (1.25, 0.625, 2.5), where all variables are in balance and remain constant. It is advisable to initiate all simulations at this point and then to introduce an intervention.

For all simulations in the following sections, the reader is strongly encouraged to predict the effect of each proposed drug treatment on *X*_3_, as well as also on *X*_1_ and *X*_2_, before the simulation is executed.

## Inhibition of influx

The pharmaceutical task is to lower the level of *X*_3_. The situation is simple, but it is useful to use the model (1) for quantitative explorations. A rather obvious idea is the introduction of a drug that inhibits the input step (Supplementary Section S2). The drug affects just the first term of the first equation:

$$\frac{dX_{1}}{dt}=Input\;\cdot {\left(Drug+1\right)}^{-1}-r_{1}{\;E\;X}_{1}$$ (2)

**FIGURE 2:**
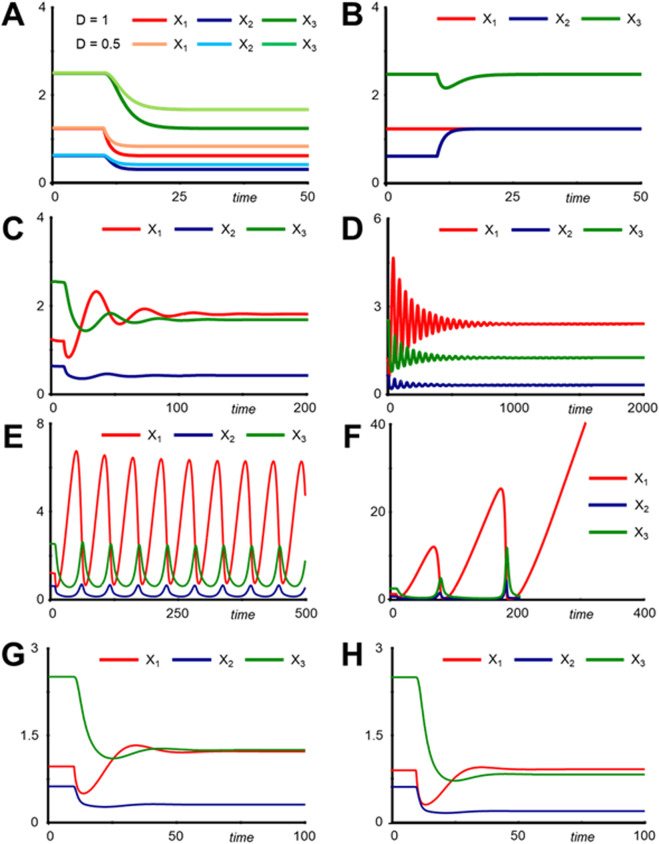
Simulation results with the pathway model in Figure 1A. **(A)** Inhibition of the influx by a drug with dose *Drug* = 1 leads to a lowering of all three metabolites. A lower dose (*Drug* = 0.5) yields correspondingly milder results. **(B)** Inhibiting the conversion of *X*_2_ into *X*_3_ does not permanently affect the end product. A dose *Drug* = 1 is administered at time *t* = 10. See Supplements of the effect inhibiting the enzyme. **(C)** Result of inhibiting the influx in the more complicated system (1B). Only variables of prime interest are shown (see Supplementary Figure S9 for other variables). **(D)** For a dose *Drug* = 1 of a drug inhibiting the input process, the system displays damped oscillations. **(E)** For the same set-up as in Figure 2D but with *Drug* = 1.2, the system enters stable “limit cycle” oscillations. **(F)** If the dose is increased further (*Drug* = 1.4), all variables disappear, except for *X*_1_, which continues to accumulate. **(G)** Implementing an isozyme converting *X*_1_ into *X*_2_ would be a good solution. **(H)** An inducer of gene *G* would also offer a good solution.

To explore the response of the system, we start the system at its steady state and without drug (*Drug* = 0) and then administer the drug by resetting *Drug* = 1, e.g., at time *t* = 10. The result is a success (Figure 2A), as *X*_3_ is lowered. Providing half the dose, *Drug* = 0.5 at time *t* = 10, *X*_3_ also decreases, but less. None of this is surprising. Of note is that *X*_1_ and *X*_2_ also decrease. It depends on the specifics of the application whether this “side effect” is pertinent. One notes that complete inhibition of the input would cause the pathway to cease.

## Activation of efflux

Instead of lowering the input, one could think of activating the efflux from *X*_3_ through a drug (Supplementary Section S3). This change affects the last term of the third equation:

$$\frac{dX_{3}}{dt}=1.6{\;X}_{2}-0.4{\;X}_{3\;}\cdot \left(Drug+1\right)$$ (3)

Indeed, this design also works: the level of *X*_3_ is lowered; in contrast to the inhibition scenario, *X*_1_ and *X*_2_ remain unaffected (Supplementary Figure S4).

## Inhibition of an intermediate step

How about inhibiting the conversion of *X*_2_ into *X*_3_ (Supplementary Section S4)? In this case, the second and third equations are affected:

$$\frac{dX_{2}}{dt}=0.8{\;E\;X}_{1}-1.6{\;X}_{2}\;\cdot {\left(Drug+1\right)}^{-1}$$

$$\frac{dX_{3}}{dt}=1.6{\;X}_{2}\;\cdot {\left(Drug+1\right)}^{-1}-0.4{\;X}_{3}$$ (4)

The response is now substantially different: The level of *X*_3_ decreases only temporarily and then returns to the original level after a short period of time (Figure 2B). In other words, this treatment strategy does not work.

Similarly, inhibiting enzyme *E* lowers the end product only temporarily (Supplementary Section S4). Furthermore, these two treatments have the side effect of increasing the levels of either*X*_2_ or *X*_1_, respectively. Whether these increases would clinically be of concern depends on the specific situation. For instance, a problem could ensue if *X*_1_ and/or *X*_2_ were toxic. Also, increased levels of intermediates create a certain degree of metabolic burden (Snoeck et al., 2024).

## Model with *TF* and *G*

Now suppose that *X*_3_ triggers the activation of a transcription factor *TF*, which governs the expression of gene *G* that codes for enzyme *E* (Figure 1A; Supplementary Section S5). The model now involves two more variables (*TF* and *G*). Furthermore, *E* is now a dependent variable (Voit and Kemp, 2025) that can change over time and is therefore represented with a differential equation. The equations describing the system are:

$$\frac{dX_{1}}{dt}=Input-0.8{\;E\;X}_{1}$$

$$\frac{dX_{2}}{dt}=0.8{\;E\;X}_{1}-1.6{\;X}_{2}$$

$$\frac{dX_{3}}{dt}=1.6{\;X}_{2}-0.4{\;X}_{3}$$ (5)

$$\frac{dTF}{dt}=a_{4}\;X_{3}^{2}-0.5\;TF$$

$$\frac{dG}{dt}={\;a}_{5}\;TF-1.5\;G$$

$$\frac{dE}{dt}={\;a}_{6}\;G-0.5\;E$$

For consistency, we use the same settings as before and define the activation parameters as *a*_4_ = *a*_5_ = 0.5 and *a*_6_ = 0.25. The power 2 associated with *X*_3_ in the equation of TF signifies a strong effect of *X*_3_ on the activation of TF (see (Voit, 2013) and Supplementary Section S5). The steady state is roughly the same for *X*_1_, *X*_2_, *X*_3_ and now also includes *TF*, *G*, and *E*: (*X*_1ss_, *X*_2ss_, *X*_3ss_, *TF*_ss_, *G*_ss_, *E*_ss_) ≈ (1.2, 0.625, 2.5, 6.25, 2.08, 1.04). We explore the same treatments as before.

## Drug interventions

As before, we initiate the system at its steady state and start administering a drug (*Drug* = 0.5) that inhibits the influx at time *t* = 10 (Supplementary Section S6). In stark contrast to the simple model, *X*_3_ does not decrease gradually, but the variables start to oscillate in a damped manner, before settling down to a new steady state. Reaching this state, *X*_3_ is not as low as for the simpler pathway (Figure 2C; Supplementary Section S9). Moreover, *X*_1_ ends at a much higher level. One could surmise that a higher increase might reduce *X*_3_ further. Alas, *Drug* = 1 leads to stronger oscillations that take much longer to settle down (Figure 2D; Supplementary Section S10). For *Drug* = 1.2, the oscillations become permanent, with constant amplitude, forming a so-called stable limit cycle (Figure 2E; Supplementary Section S11). For even higher doses, e.g., *Drug* = 1.4, the system spirals out of control: *X*_2_ and *X*_3_ disappear altogether and *X*_1_ accumulates without ceasing (Figure 2F; Supplementary Section S12). Of course, in an actual situation, the organism would not allow unlimited increases, but the important aspect is that the system ceases to function properly.

Thus, different dosing regimens yield surprising outcomes: The system response moves from no effect (*Drug* = 0) to damped oscillations (*Drug* ≈ 0.5), persistent limit-cycle oscillations (*Drug* ≈ 1.2) and, ultimately, system failure (*Drug* ≈ 1.4). Why is this happening? It is impossible to pinpoint one or two key components of the system that cause this situation. Rather, it is the entire regulatory structure of the system and its parameterization that yield this “emerging property,” which is a somewhat mysterious hallmark of systems biology (e.g., Chapter 9 of (Voit, 2016)). Superficially, the drug causes oscillations, and if *X*_3_ approaches 0 during one of these oscillations, the activity of *E* stops, ending the conversion of *X*_1_ to *X*_2_; as a consequence, *X*_1_ accumulates and *X*_2_ disappears.

Activation of the efflux from the system yields similar results (Supplementary Section S7), leading again to damped or stable oscillations or causing the depletion of *X*_3_. The combination of inhibition of influx and activation of efflux does not remedy the situation either (Supplementary Section S7). Finally, similar to the simplified pathway, drugs targeting the intermediate steps, the enzyme, or the transcription factor are ineffective (Supplementary Section S8).

## Design of a circuit that circumvents earlier problems

It is difficult to diagnose why exactly the system is so difficult to control. One could surmise that the reason is the positive feedback, which is widely known to cause issues with stability. However, the particular system analyzed here has a stable steady state, just with a level of *X*_3_ that is considered too high and therefore “diseased.”

If simple parameter changes, induced by drugs, are ineffective, is it possible to solve the problem by modulating the “circuit design” of the existing system, e.g., by employing tools of synthetic biology? For instance, would it be feasible to induce specific genes or develop antibodies that would tie up excess material? In line with our experience with the system so far, these types of questions are difficult to answer with intuition alone but are well suited for exploration with methods of systems biology.

A first target to explore might be the accumulation of *X*_1_, which occurs concomitantly with the disappearance of *X*_3_. Would it help to remove a portion of *X*_1_? The simplest strategy is to siphon off material (Supplementary Section S9). Indeed, this strategy seems to be viable (Supplementary Figure S22): *X*_3_ is substantially lowered. Surprisingly, this strategy turns out to be sensitive to variations in inputs, which may lead to the disappearance of *X*_2_ and *X*_3_ (Supplementary Figure S23).

As an alternative to removing material from *X*_1_, one could consider an isozyme that converts *X*_1_ into *X*_2_. If such an isozyme already existed, but with low capacity, one could possibly activate it through some pharmaceutical intervention. Otherwise, one could possibly manipulate the genome with methods of synthetic biology. The corresponding model is easy to set up (Supplementary Section S10). With the activation of such an isozyme, and with the additional inhibition of the input flux, the system responds exactly as we hoped. Thus, this circuit-redesign strategy, if implementable, would offer an excellent solution (Figure 2G; Supplementary Section S24).

As a variation on this strategy, one could explore over-expressing or under-expressing the gene coding for *E*, for instance, by means of an inducer that is independent of the transcription factor *TF*. Again, a corresponding model is easy to set up (Supplementary Section S11). Adding a drug inhibiting the input or a drug activating the efflux from *X*_3_ yields very encouraging results: *X*_3_ is substantially lowered, while *X*_1_ and *X*_2_ are only modestly affected (Figure 2H; Supplementary Section S25).

## Discussion and conclusion

The purpose of this illustration is not to solve a specific pharmaceutical problem but to demonstrate that intuition is not always sufficient for effective drug targeting and that a systems biological model analysis can be a potent tool for exploring alternative solution strategies, especially within the context of personalized medicine (Deisboeck, 2009; Kumbale et al., 2021; Voit and Brigham, 2008).

The analyzed system without feedback is easy to understand, and several strategies may be applied to achieving the goal of lowering *X*_3_. By contrast, the system with feedback via a gene expression loop causes our intuition to go astray and illustrates that formal computational analysis may be necessary for predicting the success or failure of different intervention strategies. Even a simple drug-induced change in influx or efflux yields surprising results: Weak inhibition leads to a slightly lower *X*_3_, as expected, but if the dose is increased, the system starts to oscillate and eventually fails.

While the feedback system in our example is already complex enough to evade intuition, it is obviously still very simplistic. Nature often employs secondary pathways and other means of apparent redundancy and control that equip a system with tolerance toward perturbations. Another simplification of the example is that the generic “drug” has a constant value, which is unrealistic. Also, a physiological feedback loop may incur substantial time delays, which are ignored here. This is especially the case for longer pathways and for physiological systems beyond pure metabolism. For instance, the feedback in the β-lapachone example is rather slow, as transcriptional regulation occurs on the scale of hours or even days (Panieri and Saso, 2021; Panieri et al., 2020). To mimic this reality, one could include more intermediary variables or introduce artificial delays (Mocek et al., 2005; Voit et al., 2022). Finally, different patients presumably exhibit differences in critical parameter values (Davis et al., 2019; Kumbale et al., 2021). A more sophisticated computational model could account for these issues, but it is the intentional simplicity that crystallizes key features of the system and its responses.

According to the model analysis, targeted changes to the system’s “circuit design” provide desirable solutions. Two efficacious strategies are: (1) the implementation or activation of an isozyme that works in parallel with *E*; and (2) the induction of expression of the gene coding for *E* that is independent of the transcription factor involved in the original system. While the model analysis suggests such solution strategies, the actual implementation of one strategy or the other falls within the realms of experimental pharmacology and synthetic biology.

## Data Availability

The original contributions presented in the study are included in the article/Supplementary Material, further inquiries can be directed to the corresponding author.
