# Supplementary material for: Systems biology can provide guidance to synthetic biology in the pursuit of new drug targets
Source: Front Pharmacol. 2026 Mar 26;17:1770107. doi: 10.3389/fphar.2026.1770107 (PMC13062750; doi:10.3389/fphar.2026.1770107)
Supplement: Supplementary file 1 [file DataSheet1.pdf]

Supplements

Systems Biology can Provide Guidance to Synthetic Biology in the Pursuit of New Drug Targets

Eberhard O. Voit

The Supplements contain additional details regarding the model set-up and some further simulation results. The sections are ordered according to the main article.

Section S1 (Model Set-up)

As explained in the text, the base scenario is a patient who has an elevated level of metabolite X_3, which is generated through a linear pathway consisting of an *Input,* precursors X_1 and X_2, and an enzyme E catalyzing the conversion of X_1 into X_2 (**Figure 1A** in the Text). The task is to lower the level of X_3.

Initially ignoring the activation of a transcription factor and the corresponding expression of the gene that codes for the enzyme converting X_1 into X_2, the diagram is simplified to

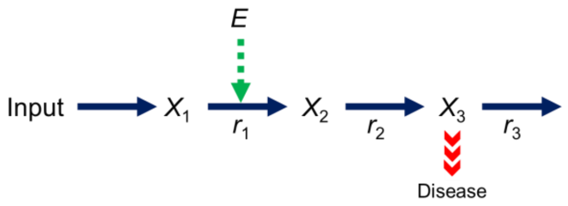

Fig. S1. Linear metabolic pathway with input. Enzyme E catalyzes the conversion of X_1 into X_2. The quantities r_1, r_2, and r_3 are the rates of conversion in each step. X_3 is a risk factor for a disease.

The model equations are presented in Eq. (1) of the Text. The enzyme is considered an independent variable, which is constant.

Numerous software packages are available to solve the ordinary differential equation models in this article numerically; they include Matlab, Mathematica, Python, Julia, R, C++, Fortran, and many others. Here, the easy-to-use, free software PLAS [1] was used.

It is typically a good strategy to start a simulation at the steady state of the system (see Text) and then to introduce a manipulation. This steady state is obtained by setting the three differential equations equal to zero, indicating that they do not change over time. Using $Input = 1$, $E = 1$, the equations are linear and solved with methods of linear algebra.

Section S2 (Inhibition of Influx)

To model the effect of a drug, we introduce the term $(Drug + 1)$, which affects a targeted process within the system in a multiplicative manner. The quantity 1 is added so that the effect of the multiplier is neutralized for a drug dose of $Drug = 0$. If the drug is an inhibitor, the multiplier becomes $(Drug + 1)^{-1}$ so that the reduction of the inhibited process becomes stronger for larger drug doses. These settings are commonly employed for metabolic systems analyses [2, 3].

In the corresponding diagram, the drug effect is represented with a blunted line (inhibitor) or with an arrow in the case of an activator (Section S3). For instance, inhibition of the input process (as analyzed in the Text) is shown in **Figure S2**.

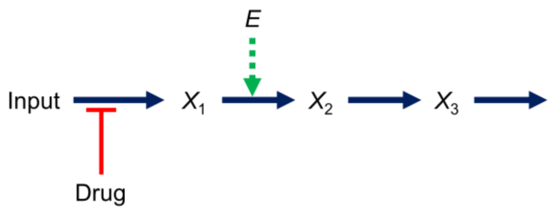

Figure S2. Inhibition of the input process of a linear pathway by a drug.

Starting at the steady state, all variables remain at their initial values, until the drug is administered as an intervention.

It is noted that if the conversion of X_1 into X_2 follows a Michaelis-Mention process, responses to increases in input flux may become saturated, which could in some cases explain idiosyncratic responses to the drug.

Section S3 (Activation of Efflux)

Increasing the efflux from X_3 (**Figure S3**) affects only the last equation of the system (Eq. (3) in the Text); the first two equations are unaffected.

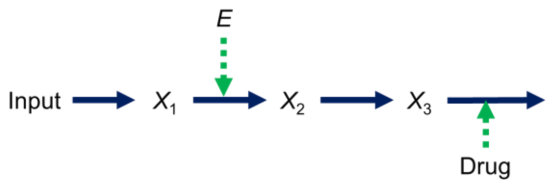

Figure S3. Activation of the efflux of a linear pathway by a drug.

Simulation confirms that the design works: the level of X_3 is lowered (**Figure S4**). In contrast to inhibiting the input, X_1 and X_2 are unaffected.

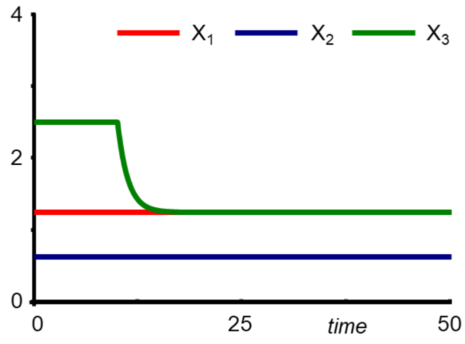

Figure S4. Consequence of activating the efflux from the system with dose $Drug = 1$ at time $t = 10$.

Section S4 (Inhibition of an Intermediate Step)

Another strategy could be to use a drug inhibiting the conversion of X_2 into X_3 affects the 2nd and 3rd equations (Eq. 4 in the Text; **Figure S5**); in this case, the first equation is not affected. In contrast to the two previous scenarios, this strategy does not work: after a slight temporary decrease, X_3 returns to its original steady-state value and X_2 increases (**Figure 2B** of the Text).

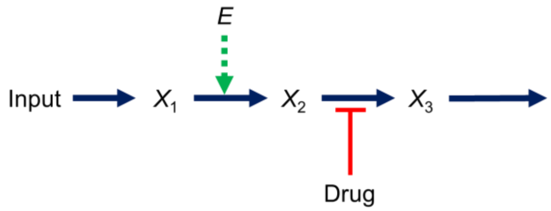

Figure S5. Inhibition of the conversion of X_2 into X_3 by a drug.

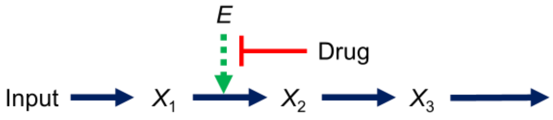

Figure S6. Inhibition of the enzyme E, which catalyzes the conversion of X_1 into X_2.

Inhibiting the enzyme E (**Figure S6**) leads to the model

$$\frac{dX_1}{dt} = Input\ -\ 0.8\ E\ X_1 \cdot (Drug + 1)^{-1}$$

$$\frac{dX_2}{dt} = 0.8\ E\ X_1 \cdot (Drug + 1)^{-1} -\ 1.6\ X_2$$

$$\frac{dX_3}{dt} = 1.6\ X_2\ -\ 0.4\ X_3$$

$$Drug = 0;\ \ at\ t = 10, set\ Drug = 1$$

Again, this strategy is not efficacious (**Figure S7**). Furthermore, this treatment has the side effect of increasing X_1 considerably; whether this increase would be of concern depends on the specific situation.

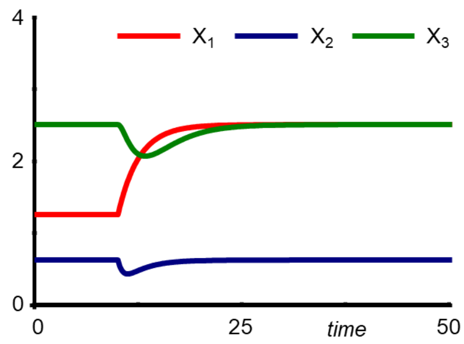

Figure S7. Inhibiting enzyme E ($Drug = 1$) lowers the end product only temporarily and leads to an increase in X_1.

The speed with which a drug disappears from plasma is roughly 4 to 5 half-lives of the elimination process, according to first-order pharmacokinetics [4]. For a metabolic (or physiological) pathway, a similar estimate may hold but the situation is more complicated. First, all variables regain a state close to the steady state at different times. Second, the time it takes to regain such a state depends on the length and the rate constants of the pathway as well as the magnitude of the perturbation. Especially in physiological systems with steps beyond metabolism, and even more so in systems with feedback, as they will be discussed next, this time period may become long, which could cause issues with toxicity and pleiotropic drugs.

Section S5 (Full Model)

The graphical representation of the pathway diagram (**Figures 1A, S8A**) seems straightforward but is not quite "clean," as X_3 does not **become** TF, but affects it, and TF does not **become** G [5].

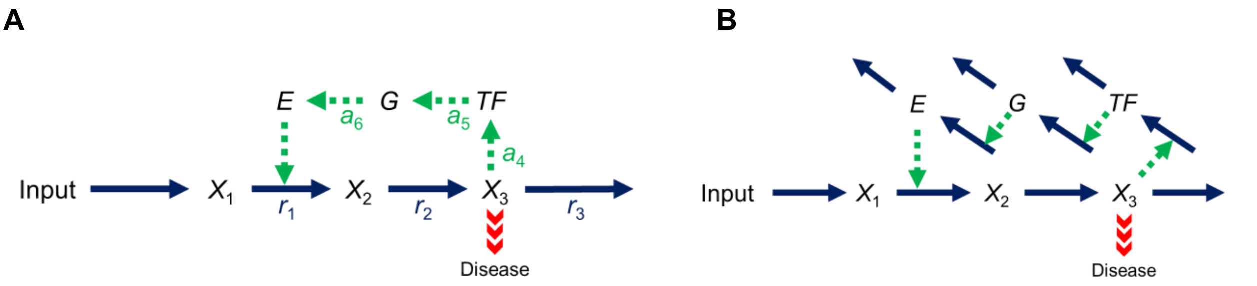

Figure S8 A. Diagram of a pathway with feedback, as presented in **Figure 1A**, in which the end product and disease risk factor X_3 triggers the generation of a transcription factor TF, which leads in the enhanced expression of gene G and the corresponding increase in the amount of enzyme E. **B.** A somewhat awkward, but theoretically better representation of the system than in **A**, because the various components are effectors of increases in other variables, rather than precursors in a metabolic sense.

This difference suggests the theoretically more appropriate representation in **Figure S8B**, which however is awkward. The mathematical difference in the associated equations is that the diagram in **Figure S8A** indicates precursor-product relationships between X_3 and TF, TF and G, and G and E, which mandate that each term with a negative sign in the precursor equation is structurally and numerically equivalent to the positive term in the product equation. For instance, the negative terms in the equations of TF and G (Eq. 5 in the Text) would have to be $a_5\,TF$ and $a_6\,G$, respectively. In contrast, they could (or could not) be different for the diagram in **Figure S8B**.

The equations are presented in the Text as Eq. (5). The power 2 associated with X_3 in the equation of TF signifies a strong effect of X_3 on the activation of TF. This formulation is in line with the tenets of the generic modeling framework of Biochemical Systems Theory [2, 3, 6-8], which is rigorously based on Taylor's approximation theory [7] and provides essentially unbiased representations [9].

Section S6 (Inhibition of Influx)

Inhibiting the influx to the full system again only affects the first term of the first equation, as in the simplified model:

$$\frac{dX_1}{dt} = Input\ \cdot (Drug + 1)^{-1} - \ 0.8\,E\,X_1$$

$$Drug = 0;\ \ at\ t = 10, set\ Drug = 0.5$$

In stark contrast to the simple model, the system now responds to the drug administration ($Drug$ = 0.5) with damped oscillations in all variables (**Figures 2C** and **S9**). For a stronger dose ($Drug$ = 1), the oscillations decrease much slower in amplitude (**Figures 2D** and **S10**). For $Drug$ = 1.2, the oscillations do no longer decrease in amplitude but instead form a so-called stable limit cycle, where the oscillations persist unless there is some intervention (**Figure 2E**). The limit cycle can be visualized differently if one plots X_3 versus X_1 (or X_2, rather than time; heavy line in **Figure S11**). All other trajectories (the time trends of the system) spiral out from the inside or in from the outside, converging to this limit cycle from both sides. As supported by the theory of differential equations, the system possesses an unstable steady state (X_{1ss}, X_{2ss}, X_{3ss}, TF_{ss}, G_{ss}, E_{ss}) = (2.64, 0.284, 1.14, 1.29, 0.43, 0.22) inside the limit cycle with two eigenvalues that have positive real parts.

For an even higher dose, $Drug$ = 1.4, the system fails, with X_2 and X_3 disappearing altogether and X_1 accumulating without end. In reality, the organism would limit the increase in X_1, but the point here is that X_3 disappears (**Figure 2F** and **S12**).

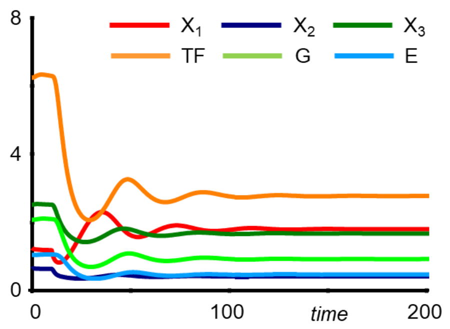

Figure S9. Inhibition of influx in the more complicated system in Eq. (5) of the Text.

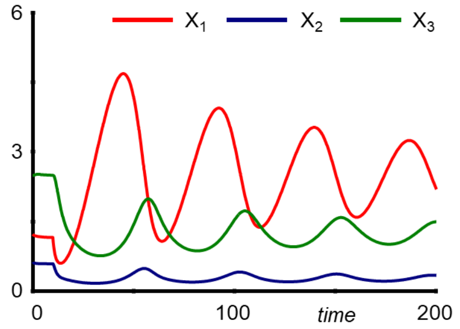

Figure S10. For a dose of $Drug = 1$, the system displays damped oscillations that are slower and stronger.

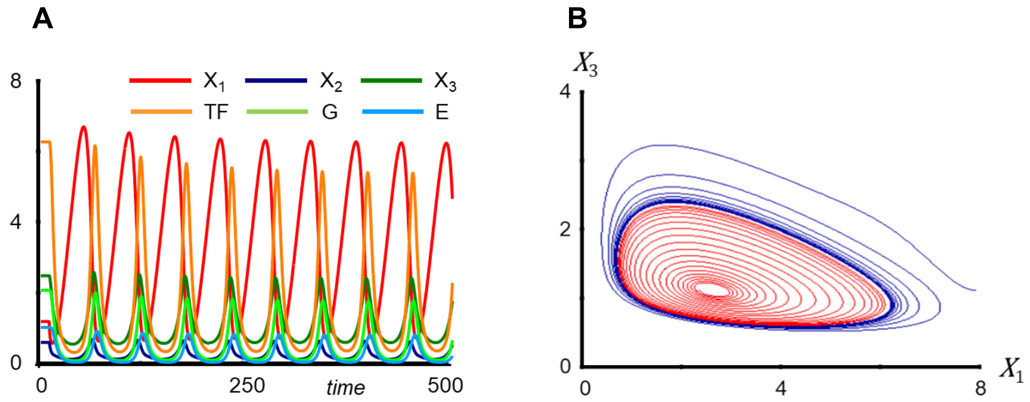

Figure S11. A. For $Drug = 1.2$, the system enters stable "limit cycle" oscillations. **B.** Starting the simulation with all variables at the unstable steady state, except for X_1, which is started at 3 times the value of X_{1ss} (2.64 · 3, blue) or 0.9 times the value of X_{1ss} (2.64 · 0.9, red), the trajectories approach the limit cycle (dark blue) from the inside or the outside, respectively.

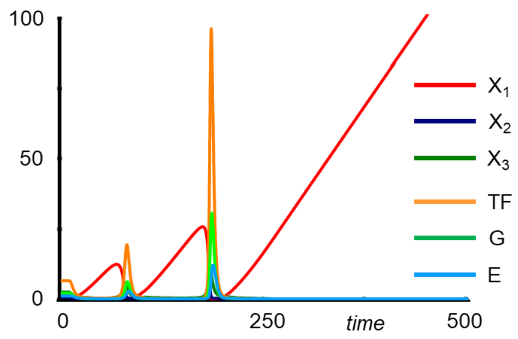

Figure S12. For *Drug* = 1.4, the system oscillates in two waves before all variables crash, except for X_1, which keeps on accumulating.

Section S7 (Activation of Efflux)

As in the simpler situation without feedback, activation of the efflux from X_3 pertains exclusively to the equation for X_3:

$$\frac{dX_3}{dt} = 1.6\, X_2 - \; 0.4\, X_3 \cdot (Drug + 1)$$

For relatively weak activation, the system enters damped oscillations (*Drug* = 0.5; **Figure S13**). The ultimate steady-state level of X_3 decreases from 2.5 to 1.67.

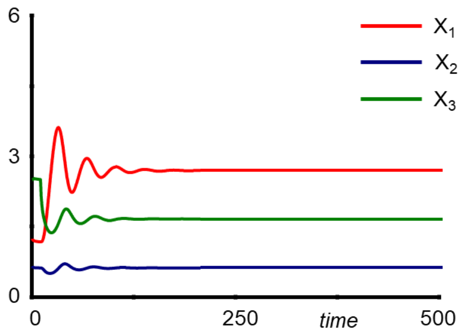

Figure S13. For a relatively weak activation (*Drug* = 0.5), the system enters damped oscillations, leading to a steady-state concentration of X_{3ss} = 1.67, which is noticeably down from 2.5.

For a stronger activation (*Drug* = 1), the response is again drastically different from the response in the simpler system. The system enters stable (limit cycle) oscillations (**Figure S14**), and for an even stronger dose (*Drug* = 1.2) the system fails, with oscillations growing in amplitude, before X_2 and X_3 are entirely eliminated and X_1 accumulates (**Figure S15**).

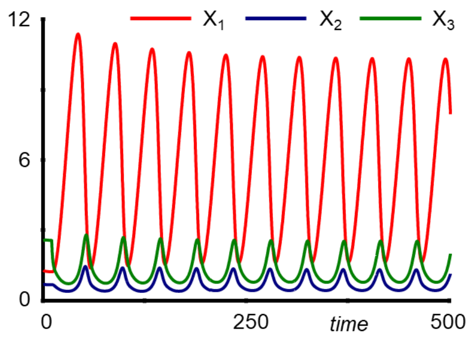

Figure S14. Stronger activation ($Drug = 1$), causes stable oscillations in all variables. Here, only X_1, X_2, and X_3 are shown.

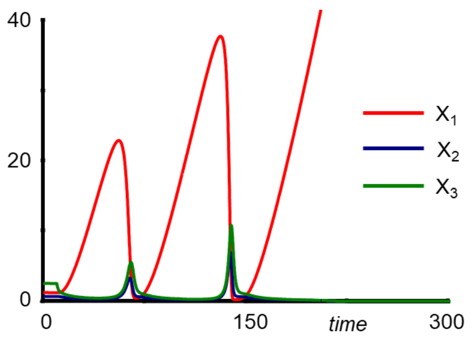

Figure S15. Under even stronger activation ($Drug = 1.2$), X_2 and X_3 disappear, which X_1 accumulates.

If activation of the efflux does not help, maybe one could explore inhibition of the efflux. The third equation then reads

$$\frac{dX_3}{dt} = 1.6\,X_2 - 0.4\,X_3 \cdot (Drug + 1)^{-1}$$

$$Drug = 0; \;\; at\ time\ t = 10, set\ Drug = 0.5$$

Now all system variables do reach a steady state, but the concentration of X_3 is even higher than without the drug ($X_{3ss} = 3.75$ instead of 2.5; **Figure S16**). Note that there are no oscillations.

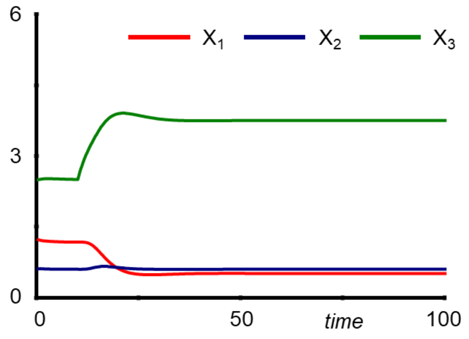

Figure S16. Inhibition of the efflux ($Drug = 0.5$) leads to an increased level of X_3.

All variables, including *TF*, *G*, and *E* (not shown) reach a steady state.

Simultaneously inhibiting the influx and activating the efflux affects the first and third equations:

$$\frac{dX_1}{dt} = Input \cdot (Drug1 + 1)^{-1} - 0.8\,E\,X_1$$

$$\frac{dX_3}{dt} = 1.6\,X_2 - 0.4\,X_3 \cdot (Drug2 + 1)$$

$$Drug1 = 0; \;\; at\ time\ t = 10, set\ Drug1 = 0.25$$

$$Drug2 = 0; \;\; at\ time\ t = 50, set\ Drug2 = 0.25$$

Using for simplicity the same low dose for both drugs, this strategy indeed works, as the steady-state concentration of X_3 decreases from 2.5 to 1.6 (**Figure S17A**). The situation is reminiscent of prescribing multiple drugs for remedying a single symptom. For instance, high blood is routinely treated with a combination of, *e.g.*, chlorthalidone, amlodipine and lisinopril. This strategy permits lower doses of each drug and incurs less severe side effects from each [10, 11].

Unfortunately, the system here is sensitive to increases in input. For instance, if *Input* is increased by 25%, from 1 to 1.25, the system enters stable oscillations, and for *Input* = 1.4 at time t = 200, when the system has reached its steady state, X_2, X_3 converge to 0, while X_1 accumulates (**Figure S17B**).

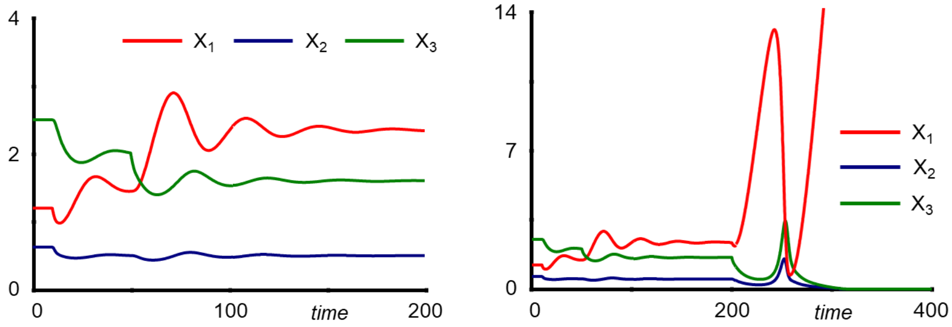

Figure S17. A. Combining inhibition of influx and activation of efflux (*Drug1* = *Drug2* = 0.25 at time t = 10) successfully lowers the level of X_3. **B.** The system is sensitive to changes in input. For instance, for *Input* = 1.4 at time t = 200, X_2, X_3 converge to 0, while X_1 accumulates.

Section S8 (Inhibition of an Intermediate Step)

In the simpler system without feedback, inhibition of the conversion of X_2 into X_3 did not help. Does it lower X_3 in the more complicated pathway? It is easy to implement the situation:

$$\frac{dX_2}{dt} = 0.8\,E\,X_1 - 1.6\,X_2 \cdot (Drug + 1)^{-1}$$

$$\frac{dX_3}{dt} = 1.6\,X_2 \cdot (Drug + 1)^{-1} - 0.4\,X_3$$

The answer is not easy to predict, but it is easily computed with changes in the corresponding equations. Alas, changes are again transient and do not lead to lasting responses (**Figure S18A**). Stronger doses (*e.g.*, $Dose = 10$) lead to under- and overshoot, as well as accumulation of X_2, but do not effect a change in X_3 (**Figure S18B**).

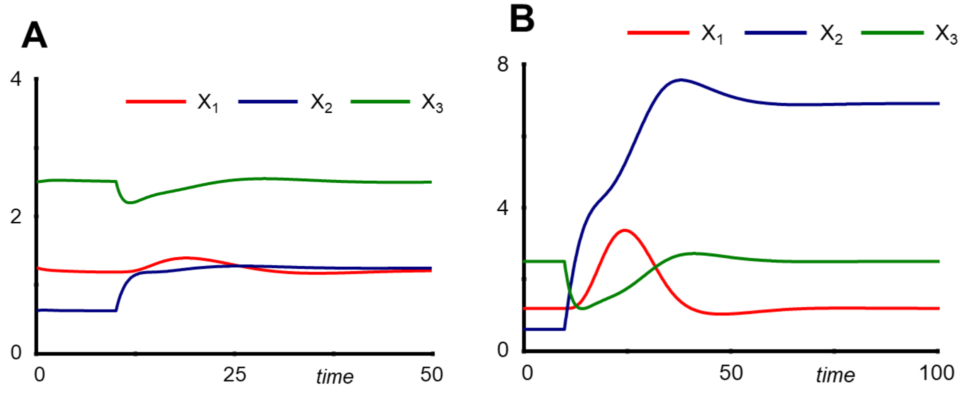

Figure S18. inhibition of the conversion of X_2 into X_3 does not lower the steady-state concentration of X_3. **A:** $Drug = 1$; **B:** $Drug = 10$.

One could instead inhibit the enzyme, arguing that less material would be moved from X_1 to X_2 per time unit. The corresponding equation is

$$\frac{dE}{dt} = 0.25\, G \cdot (Drug + 1)^{-1} \; - \; 0.5\, E$$

For a relatively low dose ($Drug = 1$), the system displays damped oscillations toward the same level as without enzyme inhibition; thus, there is no advantage (**Figure S19A**). For stronger inhibition, the oscillations become less damped and eventually follow a limit cycle ($Drug = 4$; **Figure S19B**). For even stronger doses (e.g., $Drug = 10$), the system fails (not shown).

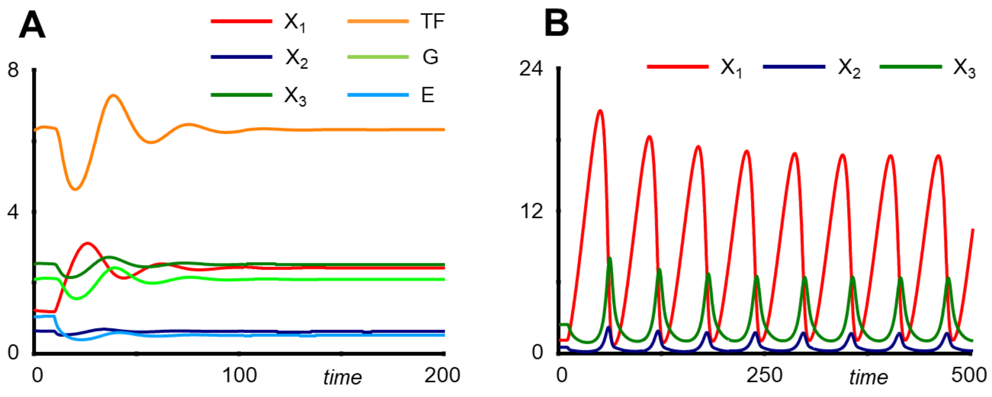

Figure S19. A: For a relatively low dose ($Drug = 1$), inhibiting enzyme E does not permanently lower the steady-state concentration of X_3. **B:** If the dose is much stronger ($Drug = 4$), the system enters a regime of stable oscillations.

Increasing the production of enzyme E

$$\frac{dE}{dt} = 0.25\, G \cdot (Drug + 1) \; - \; 0.5\, E$$

causes slight over-and undershoots, but the ultimate level of X_3 is not affected (**Figure S20**). As in the previous case, the explanation is that the change in the level of E affects the level of the precursor X_1, but does not permanently change X_3 or the overall balance between influx and efflux.

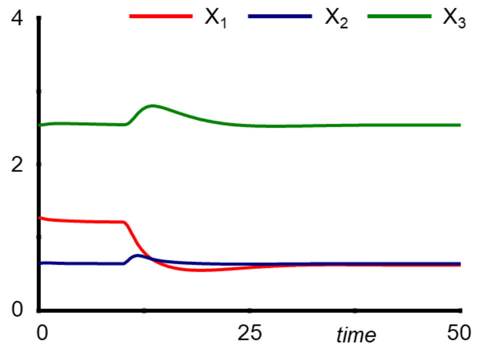

Figure S20. Increasing the production of enzyme E with a drug ($Drug = 1$) does not affect the long-term concentration of X_3.

Would it help to inhibit the transcription factor TF, for instance, with a drug, an antibody AB, or some other biologic? Mathematically, the change is readily implemented. It mandates that the equation of TF include a term that describes how the complex between TF and AB reduces the level of free (active) TF:

$$\frac{dTF}{dt} = 0.5\, X_3^2 \; - \; 0.5\, TF \; - \; r_4\, TF \cdot AB$$

Numerically, there is redundancy between r_4 and AB. So, we may set $AB = 1$ without loss of generality.

The result is disappointing. Starting at the original steady state, and then resetting r_4 from 0 to 1 at time $t = 10$, the systems starts to oscillate and then returns to the original steady state (**Figure S21A**). If the complex formation between TF and AB is made stronger ($r_4 = 2$), X_1 accumulates without end, while X_2 and X_3 decrease to zero (**Figure S21B**).

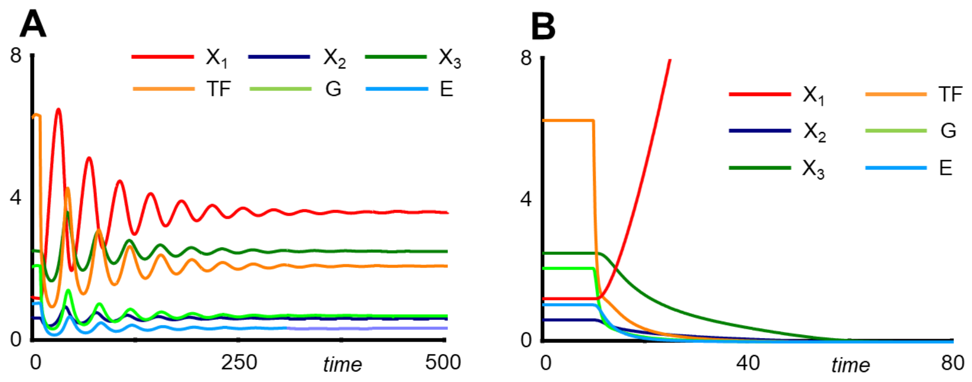

Figure S21. A. Application of an antibody ($AB = 1$) against the transcription factor TF with binding rate $r_4 = 1$ causes oscillations but no ultimate lowering of X_3. **B:** Stronger binding ($r_4 = 2$) causes X_2 and X_3 to vanish.

The explanation after the fact is similar to previous changes inside the control loop: The change in the level of TF alters the dynamics and the ultimate values of E, G and TF, but it does not change the overall balance between influx and efflux.

Section S9 (Removal of the First Pathway Metabolite)

Removal of X_1 from the system, with a rate of r_5, is easy to implement and test with the model. Only the first equation is affected:

$$\frac{dX_1}{dt} = Input \ - \ 0.8\, E\, X_1 \ - \ r_5\, X_1$$

We simulate the altered system by starting at the original steady state and then setting r_5 from 0 to a higher value at time $t = 10$. The result reveals that the additional efflux from X_1, even with a low rate ($r_5 = 0.2$) indeed causes the steady-state concentrations of X_2 and X_3 decrease (X_2 from 0.625 to 0.375; X_3 from 2.5 to 1.5). Surprisingly, X_1 increases from 1.2 to 2, even though material is siphoned off (**Figure S22**). This result is a consequence of the feedback structure of the system.

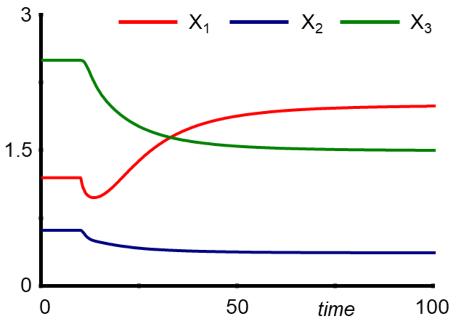

Figure S22. Removing X_1 (here with rate $r_5 = 0.2$) seems to be a viable strategy for lowering X_3.

Thus, the strategy of removing X_1 seems to be viable for lowering X_3. Unfortunately, it turns out to be rather sensitive to variations in inputs. For instance, if at some later time point (*e.g.*, $t = 100$) the

input is reduced by 10%, from 1 to 0.9 X_2 and X_3 totally disappear, while X_1 attains a much higher steady state than before (**Figure S23**).

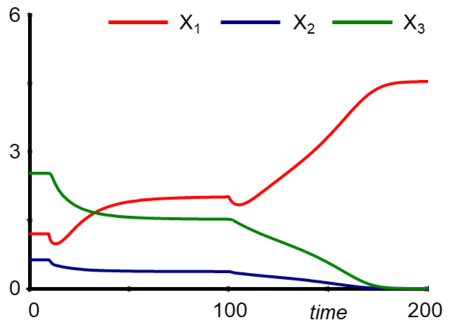

Figure S23. Starting with the original settings, we reset $r_5 = 0.2$ at time $t = 10$, as in **Figure S22**. If the *Input* is reduced from 1 to 0.75 at time $t = 100$, the system crashes and X_1 increases dramatically.

Section S10 (Implementation of an Isozyme)

Instead of removing material from X_1, one could consider the effect of an isozyme that converts X_1 into X_2 and is independent of E. Mathematically, the situation is easy to capture. It pertains to the first two equations, which become

$$\frac{dX_1}{dt} = Input - 0.8\,E\,X_1 - r_5\,X_1$$

$$\frac{dX_2}{dt} = 0.8\,E\,X_1 - 1.6\,X_2 + r_5\,X_1$$

For $r_5 = 0.2$, the new steady state of the system is (X_{1ss}, X_{2ss}, X_{3ss}, TF_{ss}, G_{ss}, E_{ss}) ≈ (0.968, 0.625, 2.5, 6.25, 2.08, 1.04). The steady-state values are unaffected, except for X_{1ss}, which is slightly lower than before (originally 1.2).

In addition to this redesign, we apply again a drug inhibiting the input:

$$\frac{dX_1}{dt} = Input \cdot (Drug + 1)^{-1} - 0.8\,E\,X_1 - 0.2\,X_1$$

$$\frac{dX_2}{dt} = 0.8\,E\,X_1 - 1.6\,X_2 + 0.2\,X_1$$

Setting $Drug = 1$ at time $t = 10$, leads to an encouraging outcome (**Figures 2G** and **S24**): All three metabolites now have lower steady-state values: (X_{1ss}, X_{2ss}, X_{3ss}) = (1.22, 0.313, 1.25). For $Drug = 2$, the values decrease further: (X_{1ss}, X_{2ss}, X_{3ss}) ≈ (1.14, 0.208, 0.833) and even higher doses do not cause undesired dynamics. Furthermore, the system is able to tolerate reasonable changes in input (*e.g.*, $Input = 0.5$, $Input = 2$) without problems (not shown). Thus, this strategy, if implementable, constitutes a very good solution.

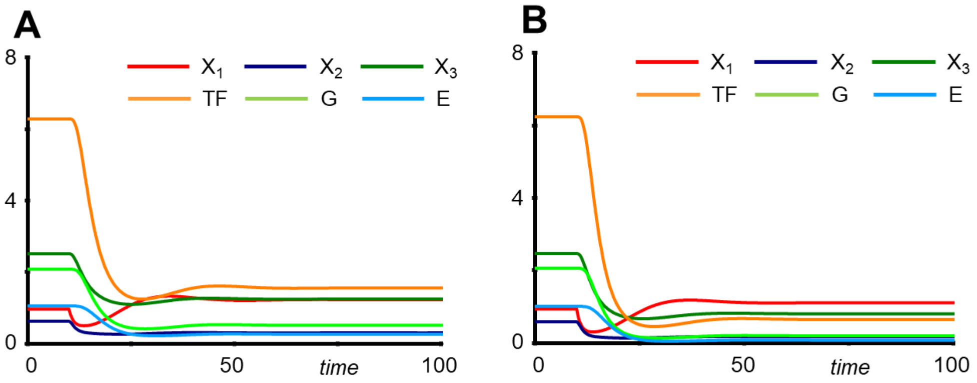

Figure S24. Implementation of an isozyme converting X_1 into X_2, combined with inhibiting the input flux, provides an excellent strategy for lowering X_3. **A.** $r_5 = 0.2$, $Drug = 1$. **B.** $r_5 = 0.2$, $Drug = 2$.

Section S11 (Implementation of an Inducer)

As an alternative strategy, one could explore over-expressing or under-expressing the gene coding for E, for instance, by means of an inducer that acts independently of TF. In this case, only the equation of G is affected:

$$\frac{dG}{dt} = 0.5\,TF \;-\; 1.5\,G + Ind$$

Again, this is a modification inside the control loop, and X_{2ss} and X_{3ss} are indeed unchanged. By contrast. X_{1ss} is decreased to 0.909 and the values of TF, G, and E are altered. Specifically, the new steady state for $Ind = 1$ is (X_{1ss}, X_{2ss}, X_{3ss}, TF_{ss}, G_{ss}, E_{ss}) = (0.909, 0.625, 2.5, 6.25, 2.75, 1.375). Similar to the previous strategy, we additionally inhibit the influx:

$$\frac{dX_1}{dt} = Input \;\cdot (Drug + 1)^{-1} - \;0.8\,E\,X_1$$

Starting at the steady state for $Ind = 1$, and changing the drug dose from 0 to 2 at time $t = 10$, we obtain a desirable result: X_3 is substantially lowered from 2.5 to 0.83; X_1 remains essentially the same (0.93), while X_2 decreases to 0.21 (**Figures 2H** and **S25**).

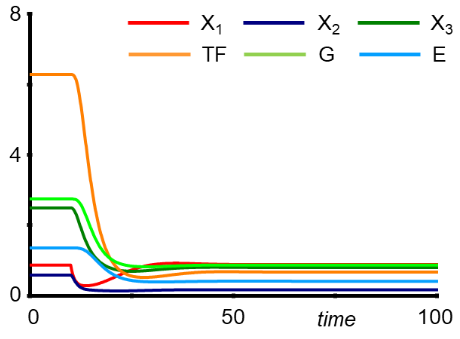

Figure S25. Inducing the gene (*Ind* = 1) that codes for enzyme *E* has the desired effect of lowering X_3, once a drug is administered that reduces the influx (*Drug* = 2).

Instead of inhibiting the influx, one could activate the efflux from X_3, as before:

$$\frac{dX_3}{dt} = 1.6\,X_2 - 0.4\,X_3 \cdot (Drug + 1)$$

$$\frac{dG}{dt} = 0.5\,TF - 1.5\,G + Ind$$

Starting again at the steady state for *Ind* = 1, and changing the drug dose from 0 to 1 at time $t = 10$, we again obtain a good result with X_3 again lowered from 2.5 to 1.25 (**Figure S26**). In this case, X_1 increases substantially, from 0.909 to 2.1, while X_2 remains the same. Thus, the inhibition and activation strategies achieve the same main goal but have different side effects.

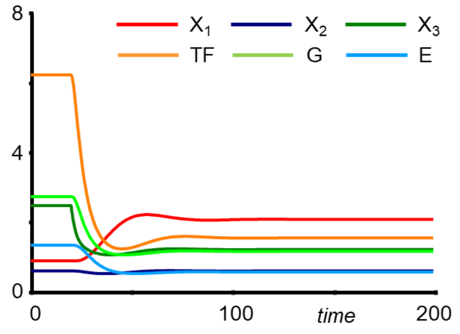

Fig. S26. Induction of gene *G* and activating the efflux from X_3 yields a good solution.

References

1. Ferreira, A.E.N., *PLAS - Power Law Analysis and Simulation.* https://arquivo.pt/wayback/20230321162314/http://enzymology.fc.ul.pt/software/plas/, 2000.
2. Voit, E.O., *Computational Analysis of Biochemical Systems: A Practical Guide for Biochemists and Molecular Biologists*. 2000, Cambridge, UK: Cambridge University Press. xii + 530.
3. Voit, E.O., *Biochemical Systems Theory: A review.* Int. Scholarly Res. Network (ISRN – Biomathematics), 2013. **Article 897658**: p. 1-53.
4. Hallare, J. and V. Gerriets, *Elimination half-life of drugs.* National Library of Medicine Bookshelf, 2025. **https://www.ncbi.nlm.nih.gov/books/NBK554498/**.
5. Voit, E.O. and M.L. Kemp, *A First Course in Systems Biology (3rd Ed.).* 2025, New York, NY: Garland Science.
6. Savageau, M.A., *Biochemical systems analysis. II. The steady-state solutions for an n-pool system using a power-law approximation.* J Theor Biol, 1969. **25**(3): p. 370-9.
7. Savageau, M.A., *Biochemical systems analysis. I. Some mathematical properties of the rate law for the component enzymatic reactions.* J Theor Biol, 1969. **25**(3): p. 365-9.
8. Savageau, M.A., *Biochemical Systems Analysis: A Study of Function and Design in Molecular Biology.* 1976, Reading, Mass: Addison-Wesley Pub. Co. Advanced Book Program (reprinted 2009). xvii, 379.
9. Voit, E.O., *The best models of metabolism.* WIREs Syst. Biol. Med. , 2017. **9**(6): p. e1391.
10. Jamerson, K.A., et al., *Initial angiotensin-converting enzyme inhibitor/calcium channel blocker combination therapy achieves superior blood pressure control compared with calcium channel blocker monotherapy in patients with stage 2 hypertension.* Am J Hypertens, 2004. **17**(6): p. 495-501.
11. Taylor, A.A., *Combination drug treatment of hypertension: have we come full circle?* Curr Cardiol Rep, 2004. **6**(6): p. 421-6.
